# How Strong are Passwords Used to Protect Personal Health Information in Clinical Trials?

**DOI:** 10.2196/jmir.1335

**Published:** 2011-02-11

**Authors:** Khaled El Emam, Katherine Moreau, Elizabeth Jonker

**Affiliations:** ^2^Department of PediatricsFaculty of MedicineUniversity Of OttawaOttawa, ONCanada; ^1^Children's Hospital of Eastern Ontario Research InstituteOttawa, ONCanada

**Keywords:** Privacy, security, passwords

## Abstract

**Background:**

Findings and statements about how securely personal health information is managed in clinical research are mixed.

**Objective:**

The objective of our study was to evaluate the security of practices used to transfer and share sensitive files in clinical trials.

**Methods:**

Two studies were performed. First, 15 password-protected files that were transmitted by email during regulated Canadian clinical trials were obtained. Commercial password recovery tools were used on these files to try to crack their passwords. Second, interviews with 20 study coordinators were conducted to understand file-sharing practices in clinical trials for files containing personal health information.

**Results:**

We were able to crack the passwords for 93% of the files (14/15). Among these, 13 files contained thousands of records with sensitive health information on trial participants. The passwords tended to be relatively weak, using common names of locations, animals, car brands, and obvious numeric sequences. Patient information is commonly shared by email in the context of query resolution. Files containing personal health information are shared by email and, by posting them on shared drives with common passwords, to facilitate collaboration.

**Conclusion:**

If files containing sensitive patient information must be transferred by email, mechanisms to encrypt them and to ensure that password strength is high are necessary. More sophisticated collaboration tools are required to allow file sharing without password sharing. We provide recommendations to implement these practices.

## Introduction

Information technology is being increasingly used in clinical trials. One recent study estimated that 41% of Canadian clinical trials are using an electronic data capture (EDC) system [1]. Researchers are also turning more to electronic medical records as a source of clinically relevant patient data, and this is fueled by their growing adoption in practice [2-6].

The data collected during clinical trials consist of sensitive personal health information (PHI). Most clinical trial data sets contain fields such as participant initials, date of birth, and gender; information about the location of the participant’s residence; and the clinical trial site where the participant is receiving treatment. This kind of information can be used to reidentify individuals [7-10]. In some cases, clinical trial data contain detailed contact information (eg, email addresses, residence address, or telephone numbers) for participants to receive reminders of upcoming visits or reminders to complete specific data collection forms.

Despite strong assurances about the safety of PHI entrusted with researchers [11] and arguments about the paucity of publicly known privacy violations in medical research [12], there have been recent publicized cases of data breaches from clinical trials [13]. Risky behaviors that can result in data breaches when handling data in clinical trials have been reported [14]:

- Engineering and mathematics graduate students were participating in a study that involved the analysis of medical images. These students did not receive sufficient education on privacy issues and how to handle PHI. Consequently, they were exchanging the personal data of subjects among themselves by email without any encryption.
- There were reported cases of study coordinators taking data home to finish some work off by saving it on to a memory stick or emailing the information to public accounts that they can access from home (eg, Gmail, Sympatico, or Rogers accounts). The data that were taken home were not encrypted.
- In one study progress notes had to be completed in an EDC system during a patient visit. There were cases where the physician or nurse completing the clinical notes mentioned the patient’s name, family physician name, sibling or parent name, or other identifying information in what they wrote. Therefore, even if the structured questionnaires used to collect data in a clinical research study exclude any identifying or potentially identifying information, patients can potentially be identified from the clinical notes that were submitted as part of the study.
- Another example involved the audit trails. If, for example, a nurse saved identifying information in the notes or comments section in an EDC form and then subsequently deletes that information, the information remains in the audit trail. In this scenario patients were reidentifiable through data that were available in the audit trails.
- In one study where an EDC was used, there were examples of password sharing (to avoid having to re-log in every time an individual was to work on a shared computer), and passwords written on notes posted on monitors were common.

Computer users are known to use email quite often to share files, and frequently as their primary file-sharing mechanism [15-18]. One survey of US enterprises found that approximately one-quarter reported that personal information (including PHI) was included in outbound emails in breach of regulations, and one-third had investigated a violation of data-protection regulations related to email within the previous year [19].

An earlier qualitative study indicated that email was often used to transfer information during Canadian clinical trials [14]. It has been noted that email is the most widely used communication mechanism in clinical trials [20]. One survey found that 50% of professionals working on clinical trials use email as their predominant method for sharing information [21], and two-thirds of clinical trials professionals responded that documents and files are exchanged with investigative sites via email [22]. Unfortunately, there are many ways for an adversary to access information sent by email, either during transmission or at its destination (see Multimedia Appendix 1).

In the United States the Health Insurance Portability and Accountability Act (HIPAA) permits the electronic transmission of PHI without encryption if the risk is deemed reasonable [23]. However, under many state breach notification laws the transmission of unprotected personal information by email may be considered a breach (see Multimedia Appendix 1). Many health care providers admit that they do not encrypt patient data when they are transmitted electronically [24]. On the other hand, some states, notably Nevada and Massachusetts, have mandated the encryption of electronic personal information in transit over public networks [25,26]. Noncompliance can subject data custodians to significant fines and penalties. It is likely that more states will follow with similar laws. Furthermore, recognizing the potential for a breach, various health systems have mandated the encryption of data transferred by email for delivering care and for research purposes [27-29].

Trials using an EDC system will have raw data available in electronic form throughout the study. Regulated trials need to comply with the US Food and Drug Administration’s (FDA’s) 21 Code of Federal Regulations (CFR) Part 11 regulations where electronic systems are used [30-35], and these include provisions for securing data to avoid tampering and ensure data integrity. Regulated trials have a higher likelihood (than unregulated trials) of being audited, and the FDA has publicized its intention of increased audits [36]. Failure to address FDA concerns expressed in warning letters could result in delays in drug and device submissions. The out-of-pocket clinical development costs for a self-originated new drug are estimated to be on average $282 million (US $467 million for capitalized costs) [37], making any delays in submissions to the FDA quite costly. Therefore, there are strong incentives by sponsors to implement reasonable security practices for such trials.

Trial participants have the expectation that their PHI will be protected by the sponsors and sites collecting data. There are also potential financial and social harms to participants if their PHI is inadvertently disclosed (see Multimedia Appendix 1).

To investigate the extent to which research staff actually protect PHI, in this paper we report on two studies: (1) a direct evaluation of one behavioral indicator of secure information management practices: the strength of passwords used to transfer encrypted electronic health information among the stakeholders in regulated Canadian clinical trials, and (2) a series of interviews of study coordinators to understand their file-sharing practices and how files are protected when shared.

## Methods

We performed two studies to investigate password strength and file-sharing practices in the context of clinical trials. Each is described below. Both study protocols were reviewed and approved by the research ethics board of the Children’s Hospital of Eastern Ontario Research Institute, Ottawa, Canada.

### Study 1: Password Strength Analysis

Over a period of 6 months the first author contacted stakeholders in 15 clinical trials known to him to determine whether they were interested in participating in this study. All of these trials used a form of EDC system for data collection and management. Stakeholders in four clinical trials were willing to participate in this study. Stakeholders were site coordinators, statisticians, monitors, and study project managers. Three studies had at least one commercial sponsor and were consequently expected to follow FDA regulations. The fourth trial did not have a commercial sponsor but was sufficiently high in profile that it received strong regulatory oversight by Health Canada.

The clinical trials that participated were not representative of all clinical trials in Canada. They were, however, likely examples of trials where the stakeholders were sufficiently comfortable with their security practices that they agreed to participate.

The stakeholders identified password-protected electronic files that were generated or created during these trials and that were sent or received by email. All files met the following criteria:

- Their format was either Microsoft Office (Microsoft Corporation, Redmond, WA, USA) or ZIP (eg, WinZip Computing, Mansfield, CT, USA) (compressed archive; the contents of the compressed files may be any other data file type, such as Word, Excel, SAS, or XML data files). All Microsoft Office files were version 2003 or earlier (for example, with the .doc or .xls file extension).
- They were encrypted or protected using a password.
- The files were sent by email between sites, data management groups, statistical analysis groups, external consultants, or central labs with at least one party in the communication within Canada.
- They were suspected or known to have PHI of the participants.

We chose these file formats because they are the most commonly used based on their market penetration. Focusing on these document types provided us with an indicator of password strengths used by PHI custodians when they are free to select whatever password they want.

Even if the EDC system used in the trial supported some form of secure file sharing, the email exchanges we obtained the files from were with individuals involved in the trial but who did not have an account on the EDC system (eg, external statisticians and information technology specialists).

In total we examined 15 files from the four clinical trials. Nine were ZIP files and the remainder were Microsoft Office documents.

We purchased two commercial password recovery tools (Visual Zip Password Recovery Master version 6.2, Rixler Software, and Accent Office Password Recovery version 2.6, AccentSoft Utilities, St Petersburg, Russian Federation) and attempted to recover the passwords. We selected those tools based on listings at the openwall.com site, usability, and recommendations from security administrators at our institutions. Using commercial tools allowed us to assess the risk from an unsophisticated adversary.

One tool would attempt to recover the password for the Word document, and the second tool would attempt to recover the password for the whole of the ZIP archive (ie, there is one password for the whole archive). The tools use a number of techniques, including a dictionary attack, common password patterns, heuristics, brute force to recover the password, and by taking advantage of known vulnerabilities.

For dictionary attacks, we enhanced the dictionaries used to include Canada-specific terms (such as city and province names and famous personality names) and other commonly used terms and passwords (see Multimedia Appendices 2 and 3). 

There are known vulnerabilities in some of the encryption methods that are used for these file types. Up to and including Word 2003, the default encryption was “97/2000 compatible.” This was an RC4 stream cipher with a 40-bit key. Because of the small key size, it would be possible to try all binary keys until one that works is found. This would not recover the password itself but would allow an adversary to access the contents of the password-protected file. Similarly, older versions of WinZip used the ZIP 2.0 encryption standard, which was considered weak. Only versions 9 and above of WinZip provide stronger encryption algorithms, such as Advanced Encryption Standard.

We used a computer running a 2 GHz dual processor with 2 GB of memory to execute the tool. The password recovery tools were allowed to run for 24 hours on each file before they were stopped.

The password recovery process was performed under the auspices of or by the stakeholder(s) themselves. Therefore, no files were transferred to any entity outside the data custodian to perform this study. The password recovery software was installed on a virtual machine and the software was run within the virtual machine on the data custodian’s equipment. The first author participated in running and monitoring the execution of the software. Each virtual machine instance, including all of the data files within it, was deleted after the analysis. We determined how many files had their password recovered during the 24-hour period.

### Study 2: Study Coordinator Interviews

We identified 121 study coordinators who responded to a previous survey [1] and were located within the Toronto-Ottawa-Montreal corridor. We randomly selected a subset of 80 coordinators and sent each an email request to participate in a 1-hour interview. Assuming that we would not be able to reach 25% of the group due to a change in contact information following the previous study (eg, change of employment, relocation), we expected our email invitation to be received by approximately 60 coordinators in total. We expected a response rate of 33% from those 60 [38]. We therefore planned for a group of 20 interviewees. The purpose of the interviews was to understand the file-sharing practices used within a recent clinical trial in which each coordinator had been involved.

The 80 selected individuals were invited by email to participate (Multimedia Appendix 4 contains the text of the invitation email). As an incentive to participate, we organized a raffle for an iPod shuffle (Apple, Cupertino, CA, USA) that took place after the interviews had been completed. All interviewees were entered in the raffle.

Depending on the location and timing, some interviews were conducted face-to-face and some were conducted by telephone. The interviews were recorded and then transcribed verbatim. The open-ended interview questions are presented in Multimedia Appendix 4. The interview guide included a series of questions on the electronic file-sharing practices used during the conduct of clinical trials. Specifically, the questions elicited information related to how research coordinators addressed security and privacy issues and why they made certain file-sharing choices during clinical trials. 

We used a general qualitative thematic approach to analyze the interview transcripts [39]. NVivo software version 8 (QRS International, Cambridge, MA, USA) facilitated the management and analysis of the data. We analyzed the data by developing a “start list” of codes based on the interview guide for the study, as well as the issues and themes that we expected to see in the data. However, recognizing that some codes would emerge or disappear during the analysis, we only used these predefined codes as starting points and embraced any new or revised issues or themes that emerged from the data.

## Results

### Password Strength Analysis

The ZIP files contained more than 2000 data files in their archive. In all cases the tools were able to recover the password, except for one file where the password could not be cracked within the 24-hour period. One of the recovered files contained coding information and dictionaries, and therefore did not have any PHI.

In all cases the recovered passwords were poorly constructed [40], with names of local locations (eg, “ottawa”), names of animals (eg, “cobra”), car brands (eg, “nissan”), and common number sequences (eg, “123”). This makes it easier for password recovery tools to guess them.

The files with recovered passwords that had PHI included Microsoft Word, Microsoft Excel, SAS, and XML (Clinical Data Interchange Standards Consortium Operational Data Model format files). They contained raw data from the clinical trials. In total, more than 10,000 patient records were in these files, and many with PHI on the subjects. For example, fields included name of study site, dates of screening and randomization, date of birth, initials, gender, and medical history.

For Microsoft Office document files, password-protecting a document is not the same as encrypting its contents [41]. Password protection controls the actions that can be performed on the document, such as who can modify a document, but the contents themselves are not encrypted. It may not always be obvious to an end user that such document protection does not protect the document contents themselves. A different program that ignores the document protections can be used to read the unencrypted contents, or they can be examined through a binary file viewer. All of the files in our sample were encrypted, but all used the default “97/2000 compatible” encryption.

Passwords on older versions of Word and Excel files are relatively straightforward to recover under certain conditions [42]. Word and Excel 2003 also have an option to use an RC4 stream cipher with a key length of up to 128 bits. A weakness in the implementation of the encryption module makes it possible for an adversary to compare two versions of a password-protected file to recover its plaintext contents [42,43]. In such a case password strength would not have affected the ability to extract the PHI. However, in our study we had only one version of each document and therefore our files were not vulnerable to this attack.

All of the ZIP files in our data set used the ZIP 2.0 encryption standard. All of the recovered passwords from the ZIP files were poor choices, and most of them were in our dictionaries or derived from words in the dictionaries (eg, ottawa followed by a digit).

### Study Coordinator Interviews

We interviewed 20 study coordinators in the Toronto-Ottawa-Montreal corridor. 

There was a marked difference between industry-sponsored trials and investigator-initiated trials. Specifically, industry-sponsored trials tended to have more formal processes in place to protect PHI and defined mechanisms for sharing data among those directly involved in the trial.

The three primary modes for sharing electronic information in the context of trials were as follows.

#### By Email

Data sent by email included mostly queries and responses to queries (eg, questions to sites about inconsistent or incomplete data for a particular patient). According to our informants, patient information was rarely encrypted when sent this way.

If PHI data files were sent by email then they were encrypted. This was used to justify the transmission of such files using an inherently insecure medium. If there was no EDC system in use in the trial or it did not support file sharing, then files were exchanged between any of the individuals and organizations working on the trial. If an EDC system that supported file sharing was deployed, then email was used to send data files to individuals who do not have accounts on the system.

#### Shared Drives

These drives were used within sites to store all trial information, including keys linking pseudonyms to patient names and Case Report Form (CRF) data. All site staff working on the trial would normally have access to the files on the shared drive. If the files were protected, the same password was often used for all of the files, and all staff who needed to access the documents would know that password. Formal processes for changing individual and shared credentials after the departure of staff were often not defined. Generally, individuals would not be taken off the access list once the trial was complete.

The file formats that we considered encourage the sharing of passwords. For example, it is not possible to assign different passwords to each individual who needs to access each of these documents. A single password is used for a document, and all individuals who need to read the document know that same password. If many documents need to be exchanged, it is not practical to have a different password for each one; therefore, often a single password is used for all documents and this password is shared among all users.

#### EDC Systems

In trials using EDC systems that support file sharing (through either an internal email system or document management features), individual patient-level data would be shared through the EDC system. The amount of access control would depend on the specific EDC system in question. If the EDC system did not support file sharing then most often email would be used.

It should be noted that, given the sensitivity of the topic, the interviewees may have held back some information. Specifically, they may not have been willing to share information about poor security practices in the trials they were participating in. Consequently, our results should be seen as an optimistic view of current practices.

## Discussion

### Summary

Previous work had indicated that password-protected files containing the PHI of clinical trial participants were being sent by email. Our initial study objective was to examine the strength of the passwords used to protect those files. Strong passwords were seen as an indicator of following good security practices in the context of clinical research.

We obtained a sample of 15 encrypted files that were sent by insecure email and were able to recover the passwords for 93% (14/15) of the files using commercial password recovery tools. Thirteen of those 14 files (93%) had sensitive health information in them. Therefore, in total 13/15 files were recovered *and* had PHI (87%). Since we were able to recover passwords using off-the-shelf tools, then it would be quite easy for an unsophisticated adversary to also do so. This result is consistent with previous research showing that health care professionals choose weak passwords to access patient data when there are no restrictions on password strength [44].

Perhaps more alarming, all of the Office and ZIP files in our sample used the default weak encryption methods. Therefore, an adversary had two different ways to extract the PHI: by attacking the weak algorithm itself or by attacking the weak password. In the current version of the WinZip tool (version 14.5), the default encryption is *still* based on the weak ZIP 2.0 standard.

At the time of this study the default applications for these file formats (ie, Microsoft Office and WinZip) did not enforce any password strengths, which means users could create any password they wished. For example, in earlier versions of WinZip that did provide password protection it was not possible to enforce a particular password strength (older versions of WinZip are still available [45]). Similarly, only recent versions of Microsoft Word have provided password strength enforcement [46]. Therefore, the passwords chosen were those that the stakeholders believed were sufficiently strong.

A follow-up interview study to examine the file-sharing practices of clinical trial study coordinators indicated that some PHI was exchanged by email that was not encrypted (eg, queries about specific patient data). Shared drives were another commonly used mechanism for exchanging files containing participant PHI. Shared drives create additional risks because, in practice, all files posted on the drive share a common password, and this common password is also shared among all stakeholders who need to access any one of the files. Sharing passwords is a violation of best-security practices. Furthermore, this goes against another best practice of limiting access to PHI to only the information that an individual needs (ie, a person who needs to access a single file should not get the password to access all files). From a regulatory perspective, it is also not possible to maintain audit trails of modifications made to files on shared drives.

### Recommendations

#### Encrypt PHI Sent by Email

Protocols can be employed to securely exchange information that was sent by email using PGP (Pretty Good Privacy) or S/MIME (Secure/Multipurpose Internet Mail Extensions) [47]. However, these tools remain quite difficult for people to use [48-50]. Furthermore, in an enterprise setting where the key management complexities are handled by a central information technology department, they are still complicated to use when communicating beyond institutional boundaries and therefore may not be suitable for distributed collaborations that cross such boundaries.

Some products bypass the key management complexities by sending a plaintext notification email to the receiver that they have received a message with a link to a secure website where they can pick up their email [51]. The receiver, however, then needs to create an account on the secure website to pick up the message. In the context of clinical trials with staff joining and leaving throughout, such an option may be workable if creating an account is simple.

Another common approach is to use the built-in password protection capabilities available in tools for common file formats (such as WinZip and Microsoft Office) and then transmit the encrypted files. Instructions for encrypting Microsoft Office and ZIP files are available [41,42,52-54]. However, caution should be exercised when using some of these tools. The default encryption standard may be a weak one. A strong encryption algorithm must be selected or set as the default.

If file encryption tools will be the main mechanism used to protect PHI, then all PHI needs to be in files, including queries and their responses.

Users may get confused between encrypting a file and protecting parts of it with a password (which does not encrypt it). Therefore, an alternative that avoids the potential for confusion is to use an external file encryption tool [55], whereby it would be clear that the whole file is being encrypted.

#### Enforce Strong Passwords

Where file encryption with passwords will be used, policies need to be put in place to ensure that strong passwords are also used. Ensuring password strength would mitigate the type of attack we describe in this paper. Standards for passwords are available [56], as well as general guidelines on email security [47] and information management security in the health care context [57].

The default applications for creating Office and ZIP files can enforce passwords, but only if the most recent versions are used, as only these have such capabilities, and they need to be set up to enforce password strength.

This needs to be augmented with privacy training for study coordinators so that they have an appreciation of privacy risks when using information technology in the conduct of trials. Training should cover procedures for the handling of electronic data, as well as providing background on the security risks of the specific technologies used in the study.

#### Minimize Password Sharing

In collaborative workflows that are common in clinical trials, current methods for file sharing are risky because they require password sharing, for example, by sharing files through email or on shared drives. It does not matter how strong a password is; if many individuals know that password then it is not a secure password.

Shared passwords make it difficult to maintain clear audit trails of individuals responsible for particular changes, which is a critical requirement in 21 CFR Part 11. For example, if multiple individuals at a site are able to view and edit an encrypted document on a shared drive because they all have the password, this would likely run afoul of the regulations because audit trails of modifications made to individual files are not maintained with shared drives.

Encryption of documents today assigns the password to the document rather than to the individual. To eliminate password sharing means creating multiple copies of each document with a unique password for every user. Commonly used contemporary tools cannot handle such additional password management complexity.

A more practical solution is to use collaboration environments, such as Microsoft SharePoint or equivalent ones. These allow the creation of repositories with different access controls for different users without the need to encrypt the documents themselves or store them on hosted email servers. Collaboration environments can also maintain detailed audit trails and version control.

#### Make File-Sharing Systems Inclusive

Modern EDC systems support secure email communications between stakeholders in the trial within the walls of the system, and some provide secure file sharing and document management mechanisms. Despite this capability, some of the stakeholders in clinical trials do not have access to the EDC system. For example, an external statistician would not normally have an EDC account and therefore may be sent a data file by email. The user base for such systems can be quite large, including individuals across multiple organizations, and these individuals change during a trial [58]. In addition, if there are multiple staff working on a trial within a single site, then they ought to all have EDC system accounts, otherwise mechanisms such as shared drives are used. Therefore, the use of an EDC system with good security practices around file sharing is insufficient insurance against inappropriate security practices unless *everyone* who needs to access files has an account on it.

File-sharing capabilities may not be embedded within an EDC system, but may also be complementing an EDC system (eg, a document management system). In such cases the same conditions noted above would need to apply.

In the future, the use of federated authentication systems could allow file sharing that is more appropriate to the workflows in clinical trials.

#### Strengthen Data Breach Notification Exemptions

It should not be taken for granted that the default file encryption algorithms used to protect PHI are strong. In fact, we found that emailing the ZIP files in our sample would be considered a data breach under the US Health Information Technology for Economic and Clinical Health (HITECH) Act because they all used the weak ZIP 2.0 standard. Furthermore, the emailing of files encrypted using the default encryption in Word 2003 and earlier would also be a breach under the US HITECH Act. Therefore, the simple technical act of encryption does not ensure that this was done effectively [59,60]. A good example illustrating this is the case of TJX Companies, the parent company of some of the largest retailers in the United States, whereby adversaries were able to crack a weak encryption algorithm and access more than 90 million credit card numbers [61,62]. Encryption exemptions should always require that the algorithms used must meet a minimal standard.

Encryption exemptions in breach notification laws should explicitly consider the strength of the passwords that are used. If, for example, a sensitive document on someone’s hacked Gmail account is encrypted and the password is “password,” then the encryption is somewhat meaningless, however strong the algorithm itself is. Based on the results of our study, it seems prudent to consider password strength in determining whether an exemption applies: it should not be assumed that encryption, even with a strong algorithm, means that it was done adequately and that the adversary would not be able to figure out the password. Some states, such as North Carolina and Oregon, recognize the risk of an adversary acquiring the decryption key or password [59], and therefore would not allow an encryption exemption from notification under those conditions.

### Limitations

Given the small number of trials from which we obtained files, broad generalization of the results is difficult. But we did expect that only trials that had good security and privacy practices would be willing to participate. We also expected that only study coordinators who were comfortable with the quality of their security practices would be willing to participate in the interviews. Therefore, the findings are expected to be biased toward those who were security-aware and were investing in protecting the data. Should this be case, then the more general state of affairs would be worse than depicted by our conservative results.

All of our data were collected from Canadian trials and Canadian coordinators. While the regulated trials from which we collected data had international sponsors and our interviewees participated in and discussed practices in international trials, our findings are specific to practices within a Canadian geography.

Our results indicate a potential privacy risk rather than an actual risk, since we do not know whether anyone has actually inappropriately accessed these files and cracked their passwords. However, this should not dilute the seriousness of the risk, since one purpose of having good password management practices is to act as a deterrent against an attack.

### Conclusions

When sharing files containing PHI in the context of clinical trials, it is critical to encrypt all PHI. However, such a practice does not provide much protection if the passwords are weak or if the passwords are widely shared. Our study indicated that the passwords used are not strong and could be compromised using a commercial password recovery tool, and that some file-sharing practices used in clinical trials promote the wide sharing of passwords among study staff.

These results suggest that stronger oversight is needed on the transfer of health information in the context of clinical trials, and better training and enforcement (technical and procedural) of good security practices.

## Multimedia Appendix 1

Background on email file sharing in clinical trials

## Multimedia Appendix 2

A manually constructed password list file

## Multimedia Appendix 3

The “npasswd” password quality-checking tool dictionary.

## Multimedia Appendix 4

Invitation and questions

## Abbreviations

CFR: Code of Federal Regulations
CRF: Case Report Form
EDC: electronic data capture
FDA: Food and Drug Administration
HIPAA: Health Insurance Portability and Accountability Act
HITECH Act: Health Information Technology for Economic and Clinical Health Act
PHI: personal health information
