# Supplementary material for: How Strong are Passwords Used to Protect Personal Health Information in Clinical Trials?
Source: J Med Internet Res. 2011 Feb 11;13(1):e18. doi: 10.2196/jmir.1335 (PMC3221339; doi:10.2196/jmir.1335)
Supplement: Supplementary file 1 [file jmir_v13i1e18_app1.pdf]

Multimedia Appendix 1: Background

1 Background on Email File Sharing in Clinical Trials

1.1 Potential Harms to Participants

Should an adversary get access to PHI, either because it was unencrypted or because they were able to decrypt it, they can commit medical identity theft. This means an imposter can obtain care or medications under someone else's identity, or make fraudulent insurance claims [1-10]. Health records have also been used by terrorist organizations to target law enforcement personnel and intimidate witnesses [11, 12]. Health record breaches can be further monetized by criminals through extortion of the data custodian by requesting large ransoms for the data [13, 14]. Personally identifying information (PII) represent critical pieces of personal data (e.g., addresses, telephone numbers, social security numbers) used to commit non-medical identity theft [15]. Medical records that contain PII can therefore be used for identity theft [16]. Furthermore, the inappropriate disclosure of health information may stigmatize individuals, making it more difficult to get certain types of insurance and jobs [17].

These provide compelling reasons why data custodians, and specifically clinical trial sites and sponsors, should ensure that data transmission, storage, and handling is secure throughout a study.

1.2 Data Breach Notification Laws and the Encryption Exemption

The inadvertent disclosure or loss of unencrypted PHI would be considered a data breach. Most states in the US have breach notification laws [18]. These require a data custodian to report a data breach to the individuals affected, state attorneys general, the media, consumer reporting agencies, and/or some other government agency.

There *are* penalties for a failure to comply with the state breach notification laws, which vary from state to state. They often permit enforcement by state attorneys general. Some states, such as Arizona, Arkansas, Connecticut and Florida, allow civil penalties. At the extreme end of penalties, some states (e.g., Arkansas and Connecticut) allow for the termination of the right to conduct business in the state. There already have been some penalties and costly settlements for failure to report a breach [19, 20].

Many jurisdictions provide a safe harbor for encrypted data in that no notification is required [21, 22]. Title XIII of the American Recovery and Reinvestment Act (the HITECH Act), has the additional requirement that the encryption must meet accepted national standards [23]. Guidance for the California breach notification law by the Office of Privacy Protection does specify standards for encryption, but these are not binding [24].

Therefore, the sharing of PHI that is not encrypted would subject data custodians in clinical trials to the breach notification requirements. These can be costly and damaging to the reputation of the sites and sponsors. For instance, individuals lose trust in the organizations that collect data from them if there is a breach [25-27] and listed corporations suffer a loss in their share price after the announcement of a security breach [26, 28-31]. After a breach, there are also costs associated with investigations, the notification itself, litigation, redress and compensation [32, 33]. Recently, regulators and courts in the US and the UK have started imposing fines on organizations after particularly egregious breaches, repeat offenses, or to set examples [34-40].

1.3 Participant Expectations When PHI is Sent by Email

Providers and patients do use insecure email to exchange PHI. The proportion of US Internet users who reported communicating with their health care provider was 10% [41], a European survey found that 4% have approached their family doctor over the Internet, and about 7% of email users in the US exchange emails with physicians or health professionals [42]. The proportion of physicians who report communicating by email with their patients varies from 3.6% to 24% [43-45]. About a quarter of patients correspond via email with family members [46]. PHI may also be exchanged electronically among patient peers [47].

Professional associations recommend obtaining an explicit patient waiver for using unencrypted email for patient – provider communication [48-51]. Under those conditions the patient has consented to the elevated risk of an adversary getting hold of their PHI. In the context of clinical trials, however, the participants do not waive their expectation that their PHI will be transmitted, stored, and handled in a secure manner.

1.4 Password Strength

A common way for encrypting files when they are shared, say through email, is to use a password. It is known that strong passwords are difficult to remember [52, 53]. Therefore, users choose memorable passwords that are also easy to crack through dictionary attacks [54, 55], use words that are familiar to them in their passwords [56], are easy for their spouses and partners to guess [52], and generally select the weakest passwords that they can get away with [57]. It has been argued that security advice, including advice on the choice of strong passwords, offers poor cost-benefit tradeoffs for users and is therefore rejected [58]. Consequently, many passwords are quite weak [57, 59-69]. For example, a recent hack exposed 32,603,387 passwords from the RockYou.com site, which provides services and applications to social networking sites like Facebook and MySpace [70]. An analysis of these passwords [71] revealed the top 20 password shown in Table 1. The top 20 passwords, all poor choices, represent around 2.5% of all passwords. Our own analysis of this data further reveals that 13,589,529 used only lower case characters, 488,499 used only upper case characters, 5,193,330 used only digits (not mixing character types makes it easier to break the password), 1,405,280 were five characters or less (it is generally recommended to have at least eight characters in a password), and 9,893,677 were six characters or less. When we used our password dictionaries [72, 73] on the RockYou.com password list in a brute force attack, we found that approximately 18%-19% of the individuals had passwords in the dictionary. Healthcare professionals are not immune from creating poor passwords [74].

Password	Count	Password	Count
123456	290,731	nicole	17,168
12345	79,078	daniel	16,409
123456789	76,790	babygirl	16,094
Password	61,958	monkey	15,294
Iloveyou	51,622	jessica	15,162
Princess	35,231	lovely	14,950
Rockyou	22,588	michael	14,898
1234567	21,726	ashley	14,329
12345678	20,533	654321	14,984
abc123	17,542	qwerty	13,856

Table 1: The top 20 most frequent passwords in the RockYou.com password file. This table is based on the analysis in [71], which removed dangling spaces from the passwords and did not account for different capitalizations (e.g., “Michael” and “michael” were treated as the same password).

1.5 Getting Access to Encrypted Files with Weak Passwords

If the passwords used were weak, and consequently easily recoverable, then encryption would not provide meaningful protection if an adversary is able to obtain those files, and then decrypt them. There are a number of ways in which this could happen.

It is possible for an adversary to intercept the files in transit. This could happen, for example, if an email is inadvertently sent to the wrong person or group. Also, some frequently used free hosted email services, such as Yahoo mail and Hotmail, do not encrypt communications between the user and the mail server, making it quite easy to intercept messages and get the files being transmitted (for example, through unprotected public wireless networks). Email messages may also be relayed through multiple servers before they reach their

destination. For example, a clinical trial site may use a commercial ISP to store and forward incoming email in case its own servers go down. In principle, any of those relays can examine the payload of the messages.

An adversary can also get access to the files once they arrive in the recipient's mailbox in a number of different ways: through inadvertent disclosure of the email file when machines are sold, donated or given to another employee [75], by getting user email account passwords through phishing schemes or other types of attack [76-78], if hardware with email files is stolen from practice offices [79], from peer-to-peer file sharing networks installed on the recipient's machine [80-83], or by law enforcement if the recipient is using a hosted email service (with servers potentially located in another country) [84, 85].

There is evidence that the secret questions used to recover or account change passwords, often used on hosted web-based email providers, are quite easy to guess by friends and spouses, acquaintances or by statistical guessing [86-88], making it possible for an adversary to reset the password and get access to the email. Furthermore, subpoenas of email in civil and criminal cases are quite common [89], and requests for data from service providers by governments are numerous (for example, Google received at least 4,287 data requests from the US government in the first six months of 2010) [90]. Some judges have ruled that government agencies are allowed to search through hosted web-based email accounts without notifying the account's owner [91].

Even if the passwords were not weak, adversaries can use other methods to obtain the encryption password, for example, through keyloggers or other crimeware that may be installed on the recipient's machine [92-94], or by using password recovery tools as we did on the current study.

1.6 Dangers of Sharing Passwords

Even strong passwords protecting encrypted files by themselves may not eliminate the risks from transferring PHI electronically. The practices for managing these passwords must also be strong, say, by not revealing or sharing them. For instance, it is known that members of the public will reveal their passwords for a chocolate bar, an Easter egg, or a cheap pen [95-102].

Generally individuals will adopt behaviors to get their work done more expeditiously and efficiently, bypassing security policies and tools that hinder that objective [65, 103]. Therefore, if sharing passwords overcomes an obstacle to getting the work done, then it would not be surprising that the passwords will be shared.

2 References

1. Dixon P. Medical Identity Theft: The Information Crime that Can Kill You. 2006; The World Privacy Forum.
2. Messmer E. Health care organizations see cyberattacks as growing threat. Network World. 2008; Available from: [www.infoworld.com/print/32801]. Archived at: [www.webcitation.org/5uxGim78f]. Accessed on: May 1, 2010.
3. Wereschagin M. Medical ID theft leads to lengthy recovery. Pittsburgh Tribune-Review. 2006; Available from: [www.pittsburghlive.com/x/pittsburghtrib/news/cityregion/s_476326.html]. Archived at: [www.webcitation.org/5uxGsaBlB]. Accessed on: May 1, 2010.
4. United States of America vs. Fernando Ferrer Jr and Isis Machado. 06-60261. 2006, United States District Court Southern District of Florida.
5. Bogden D. Las Vegas pharmacist charged with health care fraud and unlawful distribution of controlled substances. US Department of Justice, United States Attorney, District of Nevada. 2007; Available from: [http://lasvegas.fbi.gov/dojpressrel/pressrel07/healthcarefraud022307.htm]. Archived at: [www.webcitation.org/5uxH2MTsw]. Accessed on: May 1, 2010.
6. Diagnosis: Identity theft. BusinessWeek. 2007; Available from: [www.businessweek.com/magazine/content/07_02/b4016041.htm]. Archived at: [www.webcitation.org/5uxH8VEHj]. Accessed on: May 1, 2010.
7. Booz Allen Hamilton. Medical identity theft: Environmental scan. 2008; Available from: [www.hhs.gov/healthit/documents/IDTheftEnvScan.pdf]. Archived at: [www.webcitation.org/5uxHJAOcC]. Accessed on: May 1, 2010.
8. Lafferty L. Medical identity theft: The future threat of health care fraud is now. Journal of Health Care Compliance, 2007; 9(1):11-20.

9. Man accused of forging prescriptions for 15,000 painkiller pills. CBC. 2009; Available from: [www.cbc.ca/canada/calgary/story/2009/02/19/cgy-pharmacy-oxycontin-fraud.html]. Archived at: [www.webcitation.org/5uxHyiAp6]. Accessed on: May 1, 2010.

10. Bird J. The uninsured turn to fraud. Charlotte Business Journal. 2009; Available from: [http://charlotte.bizjournals.com/charlotte/stories/2009/11/16/focus1.html?b=1258347600%5E2432371]. Archived at: [www.webcitation.org/5lOoTAT1N]. Accessed on: May 1, 2010.

11. 'Dissident operation' uncovered. BBC News. 2 July, 2003; Available from: [http://news.bbc.co.uk/1/low/northern_ireland/3038852.stm]. Archived at: [www.webcitation.org/5Wd0DNdAZ]. Accessed on: May 1, 2010.

12. McGuigan C, Browne M. Hospital leak linked to witness in LVF case. Belfast Telegraph. August 2007; Available from: [www.sundaylife.co.uk/news/article2896291.ece]. Archived at: [www.webcitation.org/5Wd0LZKEG]. Accessed on: May 1, 2010.

13. Kravets D. Extortion Plot Threatens to Divulge Millions of Patients' Prescriptions. Wired. 2008; Available from: [www.wired.com/threatlevel/2008/11/extortion-plot/]. Archived at: [www.webcitation.org/5hKiktSWV]. Accessed on: May 1, 2010.

14. Krebs B. Hackers Break Into Virginia Health Professions Database, Demand Ransom. Washington Post. 2009; Available from: [http://voices.washingtonpost.com/securityfix/2009/05/hackers_break_into_virginia_he.html]. Archived at: [www.webcitation.org/5hKisjsS4]. Accessed on: May 1, 2010.

15. Parkes W, Legault T, Lawson P. Techniques of identity theft. 2007; Available from: [www.cippic.ca/documents/bulletins/Techniques.pdf]. Archived at: [www.webcitation.org/5uxIHpqBt]. Accessed on: May 1, 2010.

16. Gayer J. Policing Privacy: Law enforcement's response to identity theft. 2003; Available from: [http://cdn.publicinterestnetwork.org/assets/qGQyZZXYzhsUlwOhE9QAvw/policingprivacy2003.pdf]. Archived at: [www.webcitation.org/5uxIQFgw2]. Accessed on: May 1, 2010.

17. Nass S, Levit L, Gostin L. Beyond the HIPAA privacy rule: Enhancing privacy, improving health through research, ed. Institute of Medicine. 2009: National Academies Press.

18. Lessemann D. Once more unto the breach: An analysis of legal, technological, and policy issues involving data breach notification statutes. 2009; Howard University School of Law.

19. Clark C. Hospital Fined $250,000 For Not Reporting Data Breach HealthLeaders Media. 2010; Available from: [www.healthleadersmedia.com/page-1/TEC-256217/Hospital-Fined-250000-For-Not-Reporting-Data-Breach]. Archived at: [www.webcitation.org/5sdW86ElH]. Accessed on: September 10, 2010.

20. New York Attorney General. 540,000 New Yorkers and owner of personal data not properly notified that their personal information was at risk. 2007; Available from: [www.ag.ny.gov/media_centre/2007/apr/apr26a_07/html]. Archived at: [www.webcitation.org/5t2K9iiYy]. Accessed on: May 1, 2010.

21. Burdon M, Low R, Reid J. If it's encrypted its secure! The viability of US state-based encryption exemptions. Proceedings of the 2010 IEEE International Symposium on Technology and Society. 2010.

22. Burdon M, Reid J, Low R. Encryption safe harbours and data breach notification laws. Computer Law and Security Review, 2010; 26(5):520-534.[DOI: 10.1016/j.clsr.2010.07.002]

23. Federal Information Processing Standards Publication. Security Requirements for Cryptographic Modules. 2001; National Institute of Standards and Technology.

24. Office of Privacy Protection. Recommended practices on notice of security breach involving personal information. 2007; Available from: [www.dhcs.ca.gov/formsandpubs/laws/priv/Documents/PrivacyProtection.pdf]. Archived at: [www.webcitation.org/5uyhjhQtW]. Accessed on: May 1, 2010.

25. Becker C, Taylor M. Technical difficulties: Recent health IT security breaches are unlikely to improve the public's perception about the safety of personal data Modern Healthcare, 2006; 38(8):6-7.[PMID: 16515213]

26. Garg A, Curtis J, Halper H. Quantifying the financial impact of IT security breaches. Information Management & Computer Security, 2003; 11(2):74-83.[DOI: 10.1108/09685220310468646]

27. Ponemon L. National survey on data security breach notification. 2005; Available from: [www.ehealthinformation.ca:5051/documents/Security_Breach_Survey.pdf]. Archived at: [www.webcitation.org/5uyiAJgNm]. Accessed on: May 1, 2010.

28. Acquisti A, Friedman A, Telang R. Is there a cost to privacy breaches ? An event study. ICIS 2006 Proceedings. 2006.

29. Campbell K, Lawrence G, Loeb M, Zou L. The economic cost of publicly announced information security breaches. Journal of Computer Security, 2003; 11:431-448.

30. Cavusoglu H, Mishra B, Raghunathan S. The effect of Internet security breach announcements on market value of breached firms and Internet security developers. International Journal of Electronic Commerce, 2004; 9(1):69-104.

31. Andoh-Baidoo F, Amoako-Gyampah K, Osei-Bryson K-M. How Internet security breaches harm market value. IEEE Security and Privacy, 2010; 8(1):36-42.[DOI: 10.1109/MSP.2010.37]

32. Fourth Annual US Cost of Data Breach Study. 2009; Available from: [www.ponemon.org/local/upload/fckjail/generalcontent/18/file/2008-2009%20US%20Cost%20of%20Data%20Breach%20Report%20Final.pdf]. Archived at: [www.webcitation.org/5uyitZrR4]. Accessed on: May 1, 2010.

33. Lenard T, Rubin P. An economic analysis of notification requirements for data security breaches. 2005; Available from: [www.pff.org/issues-pubs/pops/pop12.12datasecurity.pdf]. Archived at: [www.webcitation.org/5uyj6flIy]. Accessed on: May 1, 2010.

34. Information Commissioner's Office. Information commissioner's guidance about the issue of monetary penalties prepared and issued under section 55C(1) of the Data Protection Act 1998. 2010; Available from: [www.ico.gov.uk/upload/documents/library/data_protection/detailed_specialist_guides/ico_guidance_monetary_penalties.pdf]. Archived at: [www.webcitation.org/5uyjHJvFN]. Accessed on: May 1, 2010.

35. Ornstein C. Kaiser hospital fined $250,000 for privacy breach in octuplet case. Los Angeles Times. 2009; Available from: [www.propublica.org/article/kaiser-hospital-fined-250000-for-privacy-breach-in-octuplet-case-515]. Archived at: [www.webcitation.org/5gv0laEsY]. Accessed on: May 15.

36. FSA fines Nationwide over laptop theft. ComputerWeekly.com. 2007; Available from: [www.computerweekly.com/Articles/2007/02/15/221780/fsa-fines-nationwide-over-laptop-theft.htm]. Archived at: [www.ehealthinformation.ca/blogs/fsa_fines_nationwide.mht]. Accessed on: May 1, 2010.

37. THE ASSOCIATED PRESS. Seattle company agrees to pay $100,000 HIPPA fine. Seattle Post-Intelligencer. 2008. Archived at: [www.ehealthinformation.ca/blogs/seattle_company_agrees.mht]. Accessed on: May 1, 2010.

38. OCR Shines a Harsh Light on Data Breaches. Health Data Management. 2010; Available from: [www.healthdatamanagement.com/issues/18_5/ocr-shines-a-harsh-light-on-data-breaches-40156-1.html]. Accessed on: May 2, 2010.

39. Vijayan J. Court gives preliminary OK to $4m consumer settlement in Heartland case. Network World. 2010; Available from: [www.networkworld.com/news/2010/050710-court-gives-preliminary-ok-to.html]. Accessed on: July 7, 2010.

40. Mosquera M. Civil rights office steps up health privacy enforcement. 2010; Available from: [www.govhealthit.com/newsitem.aspx?nid=73735]. Accessed on: May 13, 2010.

41. Beckjord E, Rutten L, Squiers L, Arora N, Moser R, Hesse B. Use of the Internet to communicate with health care providers in the United States: Estimates from the 2003 and 2005 Health Information Trends Survey (HINTS). Journal of Medical Internet Research. 2007; Available from: [www.jmir.org/2007/3/e20]. Archived at: [www.webcitation.org/5QOqxGt6w]. Accessed on: May 1, 2010.

42. Andreassen H, Bujnowska-Fedak M, Chronaki C, Dumitru R, Pudule I, Santana S, Voss H, Wynn R. European citizens' use of e-health services: A study of seven countries. BMC Public Health. 2007; Available from: [www.biomedcentral.com/content/pdf/1471-2458-7-53.pdf]. Archived at: [www.webcitation.org/5QOrOLxi8]. Accessed on: May 1, 2010.

43. Liebhaber A, Grossman J. Physicians slow to adopt patient e-mail. Health System Change: Data Bulletin. 2006; Available from: [www.hschange.com/CONTENT/875/875.pdf]. Accessed on: May 1, 2010.

44. Grant R, Campbell E, Gruen R, Ferris T, Blumenthal D. Prevalence of basic information technology use by US physicians. Journal of General Internal Medicine, 2006; 21:1150-1155.[PMID: 16879417]

45. Brooks R, Menachemi N. Physicians' use of email with patients: Factors influencing electronic communication and adherence to best practices. Journal of Medical Internet Research. 2006; Available from: [www.jmir.org/2006/1/e2]. Archived at: [www.webcitation.org/5QOrwfK44]. Accessed on: May 1, 2010.

46. Fox S, Fallows D. Internet health resources: Health searches and email have become more commonplace, but there is room for improvement in searches and overall Internet access. Pew Internet and American Life Project. 2003; Available from: [www.pewinternet.org/pdfs/PIP_Health_Report_July_2003.pdf]. Archived at: [www.webcitation.org/5QOoYZgvN]. Accessed on: May 1, 2010.

47. Eysenbach G, Powell J, Englesakis M, Rizo C, Stern A. Health related virtual communities and electronic support groups: Systematic review of the effects of online peer to peer interactions. BMJ, 2004; 328:1166-.[DOI: 10.1136/bmj.328.7449.1166]

48. Kane B, Sands D. Guidelines for the clinical use of electronic mail with patients. Journal of the American Medical Informatics Association, 1998; 5:104-111.[PMID: 9452989]

49. American Medical Association. Guidelines for physician-patient electronic communications. 2002; Available from: [www.ama-assn.org/ama/pub/about-ama/our-people/member-groups-sections/young-physicians-section/advocacy-resources/guidelines-physician-patient-electronic-communications.shtml]. Archived at: [www.webcitation.org/5uykNyIHX]. Accessed on: May 1, 2010.

50. Canadian Medical Association. Physician guidelines for online communication with patients. 2005; Available from: [www.cfpc.ca/uploadedFiles/Resources/Resource_Items/Health_Professionals/GuidelinesOnlineCommunicationPatients.pdf]. Archived at: [www.webcitation.org/5uylygcpK]. Accessed on: May 1, 2010.

51. The Canadian Medical Protective Association. Using email communication with your patients: Legal risks. 2009; Available from: [www.cmpa-acpm.ca/cmpapd04/docs/resource_files/infosheets/2005/com_is0586-e.cfm]. Archived at: [www.webcitation.org/5uym46XhE]. Accessed on: May 1, 2010.

52. Bunnell J, Podd J, Hendersonb R, Napierc R, Kennedy-Moffat J. Cognitive, associative and conventional passwords: Recall and guessing rates Computers & Security, 1997; 16(7):629-641 [DOI:10.1016/S0167-4048(97)00008-4]

53. Zviran M, Haga W. A comparison of password techniques for multi-level authentication mechanisms. The Computer Journal, 1993; 36(3):227-237.[DOI: 10.1093/comjnl/36.3.227]

54. Klein D. Foiling the cracker: A survey of, and improvements to, Unix password security. Proceedings of the USENIX Security Workshop. 1990.

55. Morris R, Thompson K. Password security: A case history. Communications of the ACM, 1979; 22(11):594-597.[DOI: 10.1145/359168.359172]

56. Riddle B, Miron M, Semo J. Passwords in use in a university timesharing environment. Computers & Security, 1989; 8(7):569-579.[DOI: 10.1016/0167-4048(89)90049-7]

57. Florencio D, Herley C. A large-scale study of web password habits. International World Wide Web Conference. 2007.

58. Herley C. So long, and no thanks for the externalities: The rational rejection of security advice by users. New Security Paradigms Workshop. 2009.

59. Grampp F, Morris R. The Unix System: Unix operating system security. AT&T Bell Laboratories Technical Journal, 1984; 63(8):1649-1672.

60. Stoll C. How secure are computers in the USA ? An analysis of a series of attacks on Milnet computers. Computers and Security, 1988; 7(6):543-547.[DOI:10.1016/0167-4048(88)90003-X]

61. De Alvaré AM, Schultz E. A framework for password selection. 1988; Lawrence Livermore National Laboratory.

62. Menkus B. Understanding password compromise. Computers and Security, 1988; 7(5):475-481.[Doi:10.1016/0167-4048(88)90004-1]

63. Michell C. The password predictor - A training aid for raising security awareness. Computers & Security, 1988; 7(5):471-481.

64. Spafford E. Observing reusable password choices. 3rd Usenix UNIX Security Symposium. 1992. Baltimore, MD: Purdue University.

65. Adams A, Sasse M. Users are not the enemy. Communications of the ACM, 1999; 42(12):40-46.[DOI: 10.1145/1595676.1595683]

66. Brostoff S, Sasse M. Ten strikes and you're out: Increasing the number of login attempts can improve password usability. CHI 2003 Workshop on Human Computer Interaction and Security Systems. 2003. Fort Lauderdale, Florida.

67. Sasse M, Brostoff S, Weirich D. Transforming the weakest link: A human-computer interaction approach to usable and effective security. BT Technology Journal, 2001; 19(3):122-131.[DOI: 10.1023/A:1011902718709]

68. Yan J, Blackwell A, Anderson R. Password memorability and security: Empirical results. IEEE Security and Privacy, 2004; 2(5):25-31.[DOI:10.1109/MSP.2004.81]

69. Vance A. If Your Password Is 123456, Just Make It HackMe New York Times. 2010; Available from: [www.nytimes.com/2010/01/21/technology/21password.html?hp]. Archived at: [www.webcitation.org/5mweLMMk7]. Accessed on: January 20.

70. Moscaritolo A. RockYou hack compromises 32 million passwords SC Magazine. 2009; Available from: [www.scmagazineus.com/rockyou-hack-compromises-32-million-passwords/article/159676/]. Archived at: [www.webcitation.org/5vAhkIPAo]. Accessed on: May 1, 2010.

71. Consumer password worst practices. 2010; iMPERVA.

72. El Emam K. Password dictionary. 2009; Available from: [http://download.ehealthinformation.ca/cite/attack-dictionary.zip]. Accessed on: January 5, 2010.

73. npassword password files. Available from: [http://download.ehealthinformation.ca/cite/npasswd-words.tar.gz]. Accessed on: May 1, 2010.

74. Cazier J, Medlin BD. How Secure Is Your Information System? An Investigation into Actual Healthcare Worker Password Practices. Perspectives in Health Information Management 2006; 3(8).

75. El Emam K, Neri E, Jonker E. An evaluation of personal health information remnants in second hand personal computer disk drives. Journal of Medical Internet Research, 2007; 9(3):e24.

76. Krebs B. Trove of Hotmail Passwords Posted Online. The Washington Post. 2009; Available from: [http://voices.washingtonpost.com/securityfix/2009/10/trove_of_hotmail_passwords_pos.html?wprss=securityfix]. Archived at: [www.webcitation.org/5kUwwE1va]. Accessed on: May 1, 2010.

77. Update: Phishing scheme affecting some Hotmail customers. Microsoft Corporation. 2009; Available from: [http://windowslivewire.spaces.live.com/blog/cns!2F7EB29B42641D59!41528.entry?wa=wsignin1.0&sa=900906836]. Archived at: [www.webcitation.org/5kUxgYl78]. Accessed on: May 1, 2010.

78. Fildes J. Google targeted in e-mail scam. BBC. 2009; Available from: [http://news.bbc.co.uk/2/hi/technology/8292928.stm]. Archived at: [www.webcitation.org/5kUxj1f9u]. Accessed on: May 1, 2010.

79. Pitchford R, Kay S. GP practice computer security survey. Journal of Informatics in Primary Care, 1995; September:6-12.

80. Baird M. Personal files were accessible for more than three weeks. The Western Star. 2008; Available from: [www.thewesternstar.com/index.cfm?sid=104156&sc=23]. Archived at: [www.webcitation.org/5VHxgkPgX]. Accessed on: May 1, 2010.

81. Baird M. Private information leaks onto Internet ...again The Western Star. 2008; Available from: [www.thewesternstar.com/index.cfm?sid=102020&sc=23]. Archived at: [www.webcitation.org/5VHxrI7RK]. Accessed on: May 1, 2010.

82. Long J. No Tech Hacking: A Guide to Social Engineering, Dumpster Diving, and Shoulder Surfing. 2008; Burlington, MA: Syngress Publishing Inc.

83. Preimesberger C. Cyber-criminals use p2p tools for identity theft, security analyst warns. eWeek.com. 2006; Available from: [www.eweek.com/c/a/Security/Cybercriminals-Use-P2P-Tools-for-Identity-Theft-Security-Analyst-Warns/]. Archived at: [www.webcitation.org/5v1XPR0kf]. Accessed on: May 1, 2010.

84. CBC. Lakehead University, you've got Gmail. CBC News. 2006; Available from: [www.cbc.ca/canada/toronto/story/2006/12/11/google.html?ref=rss]. Archived at: [www.webcitation.org/5WvyzvoiV]. Accessed on: May 1, 2010.

85. Avery S. Patriot Act haunts Google service. The Globe and Mail. 2008; Available from: [www.theglobeandmail.com/servlet/story/RTGAM.20080324.wrgoogle24/BNStory/Technology/home]. Archived at: [www.webcitation.org/5WvzYWHaN]. Accessed on: May 1, 2010.

86. Schechter S, Brush A, Egelman S. It's no secret: Measuring the security and reliability of authentication via 'secret' questions. Proceedings of the 2009 IEEE Symposium on Security and Privacy. 2009.

87. Podd J, Bunnell J, Henderson R. Cost-effective computer security: Cognitive and associative passwords. OZCHI '96 Proceedings of the 6th Australian Conference on Computer-Human Interaction. 1996. Hamilton, NZ.

88. Zviran M, Haga W. User authentication by cognitive passwords: An empirical assessment. Proceedings of the 5th Jerusalem Conference on Information Technology. 1990.

89. Osterman Research. Outbound email and data loss prevention in today's enterprise. 2010; Available from: [www.proofpoint.com/downloads/Proofpoint-Outbound-Email-and-Data-Loss-Prevention-2010.pdf]. Archived at: [www.webcitation.org/5ux9tNwBx]. Accessed on: May 1, 2010.

90. Google. Transparency report: Government requests 2010; Available from: [www.google.com/transparencyreport/governmentrequests/]. Accessed on: July 2, 2010.

91. Law Blog. On Gmail and the constitution, in Wall Street Journal. 2009: 29 October.

92. Zeller T. Cyberthieves Silently Copy Your Passwords as You Type. New York Times. 2006; Available from: [www.nytimes.com/2006/02/27/technology/27hack.html]. Archived at: [www.webcitation.org/5tEUmdIOS]. Accessed on: May 1, 2010.

93. Holz T, Engelberth M, Freiling F. Learning More about the Underground Economy: A Case-Study of Keyloggers and Dropzones in Lecture Notes in Computer Science. 2009. p. 1-18.

94. US Department of Homeland Security, SRI International Identity Theft Technology Council, Group A-PW. The crimeware landscape: Malware, phishing, identity theft and beyond. 2006; Available from: [www.antiphishing.org/reports/APWG_CrimewareReport.pdf]. Archived at: [www.webcitation.org/5v1Yv8J8q]. Accessed on: May 1, 2010.

95. Passwords revealed by sweet deal. BBC News. 2004; Available from: [http://news.bbc.co.uk/2/hi/technology/3639679.stm]. Archived at: [www.webcitation.org/5sgui5phZ]. Accessed on: May 1, 2010.

96. Infosecurity Europe. Office workers give away passwords for a cheap pen. 2003.

97. Infosecurity Europe. Workers give passwords to total strangers in scruples survey. 2002.

98. Gibbs M. Sweet-toothed employees willing to exchange passwords for candy. itbusiness.ca. 2008; Available from: [www.itbusiness.ca/it/client/en/Home/News.asp?id=48057]. Archived at: [www.webcitation.org/5t8ECo5Ru]. Accessed on: May 1, 2010.

99. InfoSecurity Europe. Office workers give away passwords for a chocolate bar! 2004.

100. InfoSecurity Europe. Easter eggs bypass security. 2006.

101. InfoSecurity Europe. Two thirds of workers reveal passwords for chocolate and a pretty smile. 2007.

102. InfoSecurity Europe. Women 4 times more likely to give away passwords than men for chocolate. 2008.

103. Geiger K. Cloud computing in pharmaceutical R&D: Business risks and mitigations. Current Opinion in Drug Discovery & Development, 2010; 13(3):279-285.[PMID: 20443161]
