# Supplementary material for: How Strong are Passwords Used to Protect Personal Health Information in Clinical Trials?
Source: J Med Internet Res. 2011 Feb 11;13(1):e18. doi: 10.2196/jmir.1335 (PMC3221339; doi:10.2196/jmir.1335)
Supplement: Supplementary file 4 [file jmir_v13i1e18_app4.pdf]

Multimedia Appendix 2: Invitation and Questions

This appendix includes the text of the invitations to the participants in the interviews (see Figure 1), as well as the interview guide used to conduct the interviews (see Figure 2).

Subject: File Sharing Study

Dear XX,

We are conducting an interview study to understand electronic communication practices in clinical trials. With the increasing adoption of electronic data capture technologies, our objective is to understand how information is exchanged among the different stakeholders in a trial.

We wanted to invite you to participate in this interview study on electronic communication. This is a follow up to the 2008 survey on the use of electronic data capture tools in which you had participated (published here: http://www.jmir.org/2009/1/e8).

The interview will last for a maximum of one hour and will be conducted face-to-face or by telephone. A professional interviewer will come to your site or call you at a time convenient for you to conduct the interview. Your identity and information will remain anonymous and confidential. The results of this study will be used to make recommendations on improving the efficiency and security of electronic communication in clinical trials.

This research protocol has been approved by the Children's Hospital of Eastern Ontario (CHEO) Research Institute Research Ethics Board.

To thank the participants for their involvement, we will have a raffle for an iPod shuffle once all interviews have been completed. The winner will be notified by email.

If you should have any questions about this study, simply reply to this email. Our study coordinator, XXXX, will be happy to help you.

If you are interested in participating in this interview study, please click on the following link to **accept** this invitation:

"link"

You will receive a confirmation email and someone from our team will contact you to schedule a convenient time to conduct the interview.

If you *do not* wish to take part in this interview study, please click on the following link to **reject** this invitation:

"link"

You will receive a confirmation email and will not be contacted further in regards to this study.

Thank you very much for your consideration.

<principal investigator>

Figure 1: Invitation email for the interviewees.

Clinical Trial Interview Guide

Thank you for agreeing to participate in this interview. My name is XXX and I will be conducting the interview today. The interview will last approximately 1 hour and with your consent it will be audio-taped. Is it okay if I audiotape the interview?

We are conducting this interview to understand electronic file sharing practices during the conduct of clinical trials. Our objective is to understand how security and privacy considerations are addressed in practice, and why certain file sharing choices are made.

Your answers will be kept confidential. Information that may identify you will not be included in the final report or publications. You are free to stop the interview at anytime. Only answer the questions that you are comfortable answering.

Before we begin, I would like to remind you that there is no right or wrong answers.

Questions and Points of Discussion

Please tell me about the clinical trials that you have been involved with over the past five years (e.g., # of clinical trials you have been involved with, how many trials have you been involved with?). For each trial you describe, I would like to ask you some specific questions:

- # of patients recruited
- # of sites involved
- Type of study (e.g., interventional study, observational study)
- How was data captured (e.g., electronic data capture)?
- What kinds of data (e.g., completed CRFs, data queries, data files, protocols, SOPs) were available electronically?
- What format was the data in (e.g., SAS, SPSS, CDISC, Excel)?
- Who generated the data files?
- Who had access to the data files?
- Were data files shared during the conduct of the trial? Please describe the type of sharing.
    - Internally shared (including self-sharing)
    - Shared site to site
    - Shared site to central site
    - Shared site to sponsor
    - Shared with any external party (e.g., vendor, statistician)
- How were data files shared (e.g., email, USB sticks, sending CDs with files on them, printing the contents and mailing)?
    - Why did you use a shared drive as oppose to disc or paper?
    - Was data password protected or encrypted?
- If email was used, who were the files sent to (e.g., yourself, someone else)? What type of email accounts were used to send the files (e.g., gmail, hotmail, yahoo)? Do you know where the email was going and who had access to the files?
- During the conduct of the trial were you concerned about security and privacy issues?

Figure 2: Interview guide.
